# Genomic sequencing and the urgency of early diagnosis in inborn errors of metabolism

**DOI:** 10.1038/s41390-026-05140-y

**Published:** 2026-05-27

**Authors:** K. Taylor Wild, Nancy B. Spinner

**Affiliations:** 1 Divisions of Neonatology and Human Genetics, Children’s Hospital of Philadelphia, Perelman School of Medicine at University of Pennsylvania, Philadelphia, PA USA; 2 Department of Pathology and Laboratory Medicine, Children’s Hospital of Philadelphia, Perelman School of Medicine at University of Pennsylvania, Philadelphia, PA USA; 3 Pediatric Policy Council, Washington, DC USA; 4 Academic Pediatric Association, McLean, VA USA; 5 American Pediatric Society, The Woodland, TX USA; 6 Association of Medical School Department Chairs, McLean, VA USA; 7 Society for Pediatric Research, The Woodlands, TX USA

Genetic disorders are a leading cause of neonatal mortality and lifetime disability, and affected children disproportionally utilize a high percentage of health care resources. The genomics revolution in DNA sequencing technologies and disease gene identification has enabled the rapid diagnosis of genetic disorders through genome sequencing in newborns, and increasingly in the prenatal period.^1^ This progress has led to growing recognition of the potential role of DNA sequencing in both the diagnostic setting and for newborn screening.^2^ Coupled with our increasing ability to treat genetic disease via dietary modification, drug repurposing, mutation specific therapies, enzyme replacement, and RNA therapy, we are poised to help thousands of individuals a year avoid the lifelong debilitation associated with many genetic disorders.^3–5^

In the study by Wasim et al. in this journal,^6^ the authors utilize exome sequencing successfully to identify the molecular changes underlying three cases of methylmalonic acidemia (MMA) and four cases of cystathionine beta-synthase (CBS) deficiency in Pakistan, a country that does not currently have population-wide newborn screening. As one of the most populous countries globally, Pakistan faces a substantial burden of inborn errors of metabolism, with an incidence of up to 1 in 1000 births. Of these cases, approximately 30–40% are potentially treatable, highlighting the potential to diagnose and treat hundreds of thousands of affected newborns.^7,8^ While the power of the genomic technology is undisputed, in this report, diagnoses in the reported individuals were made at 5–13 years of age for the MMA patients and 1–14 years of age for the CBS patients, too late to avoid significant disease burden. For inborn errors of metabolism such as MMA and CBS deficiency, early detection is critical for effective long-term management and significantly improves outcomes.^9,10^ For MMA, early diagnosis and treatment can decrease acute metabolic crises and early mortality,^11^ and for CBS deficiency, diagnosis in infancy can prevent complications such as intellectual disability entirely, whereas delayed diagnosis often leads to irreversible cognitive impairment.^12^

While some inborn errors of metabolism can be detected on biochemical newborn screening, a molecular diagnosis with exome or genome sequencing can improve diagnostic precision, particularly for atypical cases, and can also improve prognostication and our understanding of genotype-phenotype correlations. As the potential utility of genome sequencing technologies is being realized, several countries have initiated pilot programs using sequencing technologies alongside traditional newborn sequencing, including the United Kingdom, Japan, China, Australia, and the United States, although no country has a government-mandated newborn genomic sequencing program.^13^ Exome and genome sequencing can now be performed in hours to days and can be offered as a first-line test,^14,15^ with the promise of identifying individuals at risk for many disorders, before symptoms appear, providing the opportunity to prevent clinical complications. Thus, traditional newborn screening with biochemical assays may evolve to include genome sequencing to cast a broader diagnostic net.

The tenets of newborn screening hold that the condition should represent an important health problem with an accepted treatment, be detectable at a latent or early stage, and have a suitable, population-acceptable test. Systems must exist for accurate diagnosis, treatment, and follow-up, supported by an understanding of the disease’s natural history and clear policies on whom to treat. Screening should be cost-effective, ethically and equitably implemented, and conducted as an ongoing, adaptable process rather than a one-size-fits-all approach.^16^

With these tenets in mind, employing genome sequencing as a first-tier newborn screening test offers many potential advantages but also risks. Benefits include increased diagnostic precision for conditions already evaluated by newborn screening, with the potential to diagnose as many as thousands of additional conditions. Earlier detection of many of these conditions offers the potential for targeted and sometimes preventative therapies. While these benefits are enticing, there are also caveats to consider, including the significant number of uncertain diagnoses and the potential for overdiagnosis. Genome sequencing as newborn screening could identify conditions that may not present until adulthood and conditions with variable penetrance or expressivity that may not ever present at all, resulting in increased “patients-in-waiting” with increased family anxiety and unnecessary follow-up. Particularly for adult-onset conditions, there is also the consideration that newborns cannot consent for themselves, and parents will be agreeing for the child to have the knowledge of an adult-onset condition, or increased predisposition. As a newborn screening test, this would also lead to increased cost. While the cost of genome sequencing is decreasing and it may be cost effective to offer genome sequencing as a first-tier test to children with suspected conditions,^17^ this has not yet been studied on a population level in the United States. Having genome sequencing as newborn screening will also require increased access to genetic counseling, ensuring that parents understand the implications of testing across a spectrum of disorders, with a plan for follow-up on any relevant results. Finally, there is the important risk of increased uncertainty. Variants of uncertain significance, or variants that cannot be definitively classified as disease-causing or benign, are reported in up to 60% of pediatric patients with exome and genome sequencing,^18^ with most of these variants ultimately re-classified as benign.^19^ This creates the potential for increased family anxiety as well as increased resources to follow-up or resolve these variants. Some of these risks exist for genome sequencing in general but are amplified in the context of a newborn screening test in asymptomatic populations. To better assess the utility of newborn screening via genomic testing, studies are undersway in several resource rich countries.

Accurate and early detection of treatable inborn errors of metabolism through newborn screening can significantly reduce disease burden and healthcare costs. However, in developing nations like Pakistan, the absence of a national newborn screening program creates a critical healthcare disparity, leaving infants with inborn errors of metabolism undiagnosed until irreversible damage occurs. This stark gap highlights a dire and urgent need to establish and implement systematic newborn screening programs in resource-limited settings to ensure equitable improvement in patient outcomes globally. While newborn screening with genome sequencing may increase rates of diagnosis, this will continue to widen the potential for healthcare disparities in settings where genome sequencing is not possible. Addressing these inequities will require active engagement with international agencies such as the World Health Organization and the United Nations Children’s Fund, collaboration with international philanthropic organizations, and sustained investment from resource‑rich countries. As genomic sequencing technology continues to advance, we are uniquely positioned to unlock its full potential in newborn screening, with the ability to prevent a lifetime of disability for countless infants.
